# Gold standard for nutrition: a review of human milk oligosaccharide and its effects on infant gut microbiota

**DOI:** 10.1186/s12934-021-01599-y

**Published:** 2021-05-28

**Authors:** Shunhao Zhang, Tianle Li, Jing Xie, Demao Zhang, Caixia Pi, Lingyun Zhou, Wenbin Yang

**Affiliations:** 1 State Key Laboratory of Oral Disease, National Clinical Research Center for Oral Disease, West China Hospital of Stomatology, Sichuan University, Chengdu, 610041 Sichuan China; 2 Center of Infectious Diseases, West China Hospital of Sichuan University, No. 37 Guoxue Alley, Wuhou District, Chengdu, 610041 China; 3 State Key Laboratory of Oral Diseases, National Clinical Research Center for Oral Diseases, Department of Oral and Maxillofacial Surgery, Department of Medical Affairs, West China Hospital of Stomatology, Sichuan University, No. 14, Section 3, South Renmin Road, Chengdu, 610041 Sichuan China

**Keywords:** Human milk, Human milk oligosaccharides (HMOs), Oligosaccharides (OS), Infant gut microbiota, Bifidobacterium, Prebiotics, Infant formula

## Abstract

Human milk is the gold standard for nutrition of infant growth, whose nutritional value is mainly attributed to human milk oligosaccharides (HMOs). HMOs, the third most abundant component of human milk after lactose and lipids, are complex sugars with unique structural diversity which are indigestible by the infant. Acting as prebiotics, multiple beneficial functions of HMO are believed to be exerted through interactions with the gut microbiota either directly or indirectly, such as supporting beneficial bacteria growth, anti-pathogenic effects, and modulation of intestinal epithelial cell response. Recent studies have highlighted that HMOs can boost infants health and reduce disease risk, revealing potential of HMOs in food additive and therapeutics. The present paper discusses recent research in respect to the impact of HMO on the infant gut microbiome, with emphasis on the molecular basis of mechanism underlying beneficial effects of HMOs.

## Background

It is widely acknowledged that breastfeeding is not only an evolutionary optimized means for feeding babies since ancient time but also the gold standard for infant nutrition, and World Health Organization (WHO) stipulates that mother should exclusively breastfeed her infant for the first 6 months since birth [1–3]. On the one hand, breastfeeding offer infants nutrients needed for healthy development and growth [4, 5]; on the other hand, breastfeeding also provide infants with protection against gastrointestinal and respiratory infections, and a reduced incidence of various diseases, such as obesity, diabetes, atopy, and asthma [6–12]. Besides, breastfeeding is beneficial to both babies and their mothers [13]. With recent advances and development in analytical tools for structural characterisation, scientists are engaged in the process of identifying the composition of human milk, which is featured by abundant and diverse human milk oligosaccharides (HMOs) [14]. Back to the end of the nineteenth century, the phenomenon that bottlefed infants had a much lower survival rate and a higher chance of infection in comparison to breastfed infants, aroused the scientific interest in the composition of human milk whose positive effects benefit infant health in early life. In 1900, differences in the bacteria composition between breastfed and non-breastfed infant feces were noted, and bifidobacteria seemed to enrich in breastfed infant stool [15]. With another 50-plus years efforts on HMO research, the bifidogenic factor in human milk was identified as oligosaccharides (OS) containing polysaccharides and N-acetylglucosamine (GlcNAc) [16–18]. Nowadays, more than 200 HMOs have been identified and many beneficial effects of human milk attribute to HMOs that are believed to be exerted through interactions with the gut microbiota [19]. Among components of human milk, lactose and lipids are the main source of energy to the infants which provide the uppermost source of carbohydrates at the average concentration of 30–70 g/L [20–22]. Compared with lactose and lipids, HMOs are the third largest solid component of human milk which are only slightly hydrolyzed and eventually accumulate in infant gastric intestinal tract. HMOs are complex carbohydrates and known to function as prebiotic serving as substrates for certain gut microbes in the colon tract due to their indigestible property to the infant [23, 24]. Though well regulated, the development of a healthy host-microbe symbiosis in the newborn gastrointestinal tract, which is an extremely complex and crucial biological process, is still a highly vulnerable period [25, 26]. Breastfed infants are featured by abundant *bifidobacteria* in gut microbiota, which is considered safe and beneficial to infants [27]. Besides, HMOs are increasingly linked to protection against causative organisms, such as pathogenic bacterium, virus, protozoan parasite, and fungus.

This review presents current advances in respect to the impact of HMO on the infant gut microbiome, and the critical insight into the beneficial effects of HMOs and the mechanism behind them.

## HMOs

### Chemical structure of HMOs

**Fig. 1:**
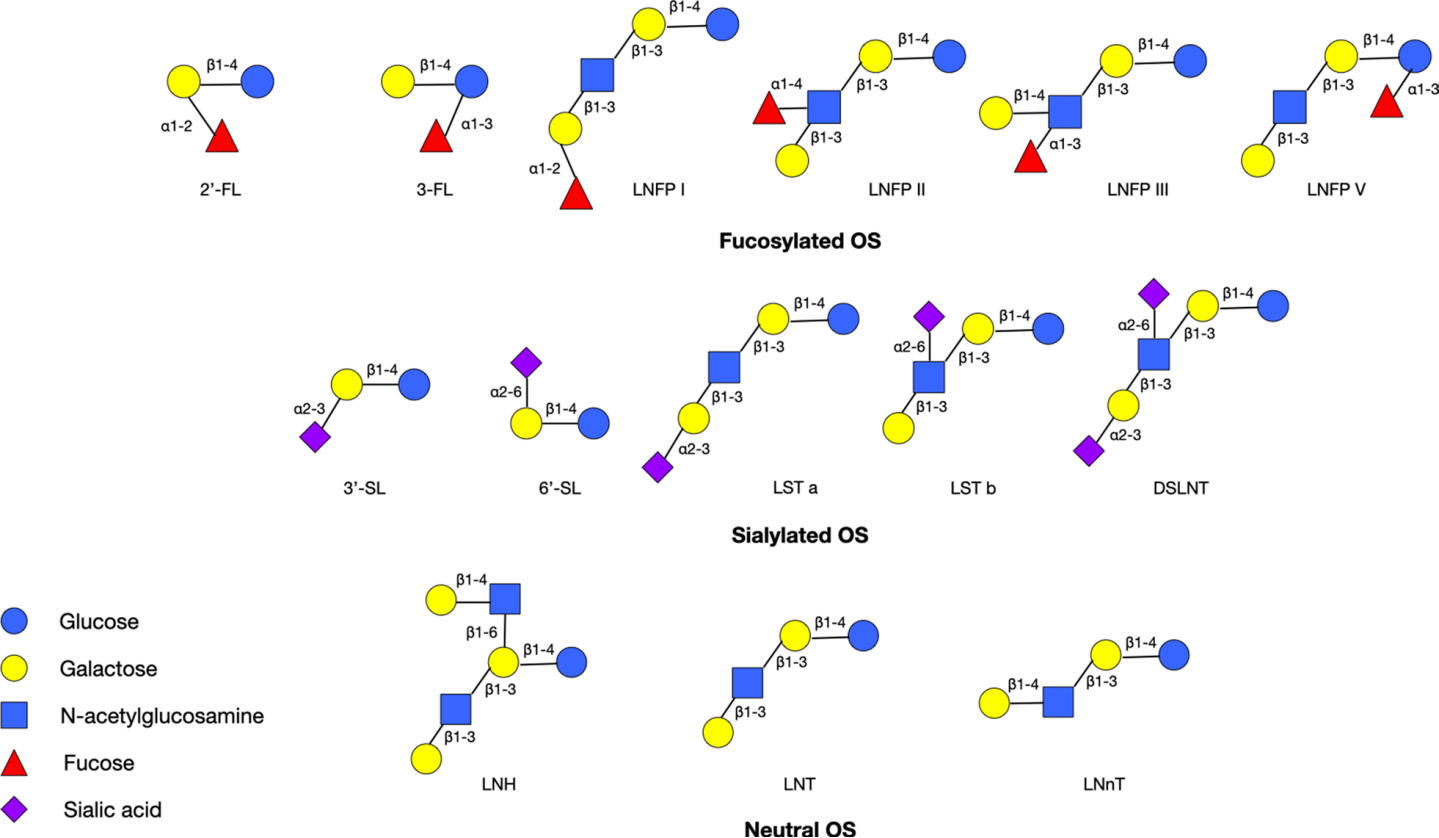
Structures of main HMOs. HMO is made up of five basic units: Sia, GlcNAc, Fuc, Gal, and Glc [28]. All HMOs contain a lactose core which can be further lengthened by LNB (type-1 chain) or N-acetyllactosamine (type-2 chain) via either *β*1–3 or *β*1–6 linkage [30]. Based on the core HMO structures are sialylated and/or fucosylated, they can be mainly classified into three groups: FucOS, SiaOS, and neutral OS [30]. Structures of the main HMO are showed

HMO is a collective terms referring to a group of various OS which is present in human milk and they are made up of five basic units including one acid monosaccharide, namely sialic acid (Sia) or N-acetylneuraminic acid; one amino sugar which is known as GlcNAc; three monosaccharides which are l-fucose (Fuc), d-galactose (Gal), and d-glucose (Glc) [28]. Although the combination of these five building blocks in diverse directions and sequences is immense, only approximately 200 different HMOs have been characterized so far and 50 compositions of HMOs are assumed to represent 99% of HMO abundance in human mother’s milk [19, 24]. All HMOs contain a lactose core (Gal*β*-1,4Glc) at the reducing end [29], which can be further lengthened enzymatically by *β*1–3 or *β*1–6 linkage to either Gal*β*1–3GlcNAc (lacto-*N*-biose, LNB, type-1 chain) or to Gal*β*1–4GlcNAc (*N*-acetyllactosamine, type-2 chain) [30]. Besides, the core HMO structures can also be decorated by Sia via *α*2–3 or *α*2–6 linkages and/or Fuc via *α*1–2, *α*1–3, or *α*1–4 linkages at the terminal positions [30]. Therefore, HMO can be mainly classified into three groups: fucosylated OS (FucOS), sialylated OS (SiaOS), and neutral OS (Fig. 1).

### Concentration of HMOs in human milk

Human milk is regarded as the golden standard for newborn babies [20] thanks to its variable composition of nutrients which contributes to the protection against pathogenic bacteria or viruses [31], prevention against bowel inflammation, and constructive modulation of the immune system response during the development of infants [32, 33]. Although composition of human milk varies from person to person, HMOs are the main nutrients benefitting newborns’ growth. HMOs are abundantly present in human milk representing about 20% of all carbohydrate in colostrum [34, 35]. The stage of lactation determines HMOs amount in human milk which varies from 20–24 g/L in the earliest human milk to10–15 g/L in mature milk on average [20, 34–36]. On the one hand, the concentration and relative abundance of non FucOS and SiaOS declined with time, but on the other hand, the relative abundance of FucOS increased despite their decreased concentration [35]. In comparison to the amount of OS in cow’s milk (up to 1 g/L in the earliest milk and 0.05–0.1 g/L in mature milk which remarkably depends on inter-breed and seasonal difference), we can find that human milk has up to 22–26 times higher levels and a higher variety of OS, indicating that the composition and structure of HMOs may be far more complicated than OS in cow’s milk [37–39].

### Endogenous synthesis of HMOs

**Fig. 2:**
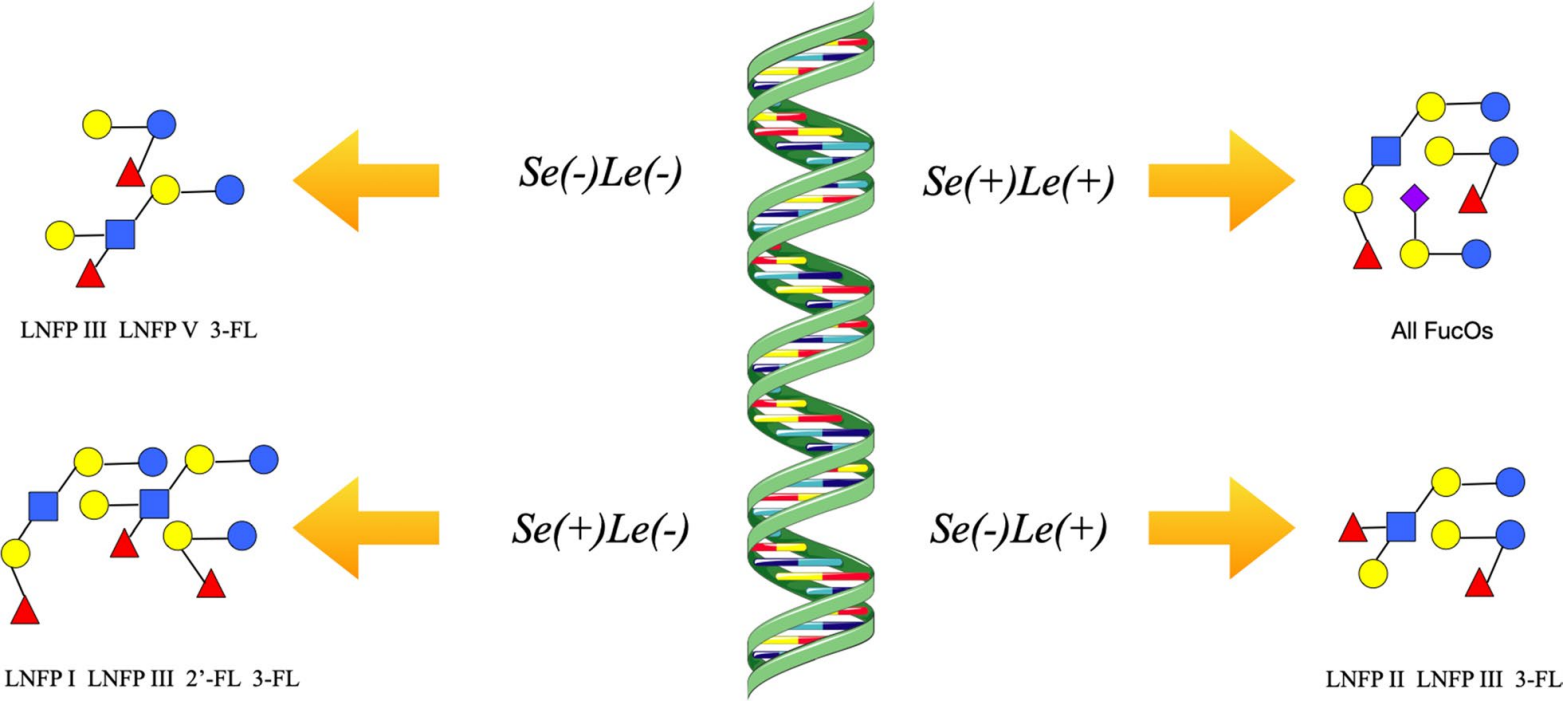
Four phenotypes of FucOS were produced by Se and Le genes [41, 44]. Se and Le genes play an important role in determining the composition of FucOS [34]. FUT2 is encoded by the first gene whereas FUT3 is encoded by the second one [40–43]. According to the activation state of genes, mothers can be classified as either positive (+) or negative (−) for both genes, where 70% are Se(+)Le(+), 20% Se(−)Le(+), 9% Se(+)Le(−), and 1% Se(−)Le(−) [36, 45–47], and phenotypes to production of FucOS and main FucOS synthesized are showed

The polymorphism of several genes contributes to wide variability of HMOs during the endogenous synthesis process. When it comes to biosynthesis of FucOS, Secretor (Se) gene and Lewis (Le) gene play important roles in encoding different fucosyltransferases to determine both the quantitative and qualitative composition of HMOs [34]. Activation of the Se gene leads to the expression of *α*1-2-fucosyltransferase enzyme (FUT2) who is responsible for lengthening the terminal Gal of the type-1 chain of HMOs by Fuc via *α*1–2 linkage [40, 41]. The Le gene allows the expression of *α*1–3/4-fucosyltransferase (FUT3) to add Fuc with *α*1–3/4 linkage to a subterminal GlcNAc of the type-1 chain of HMOs [42, 43]. According to the different expression of both Se gene and Le gene, mothers can be classified as either positive (+) or negative (−) for both genes and divided into four different groups (Fig. 2): Se(+)Le(+), Se(−)Le(+), Se(+)Le(−), and Se(−)Le(−) [41, 44], where 70% are Se(+)Le(+), 20% Se(−)Le(+), 9% Se(+)Le(−), and 1% Se(−)Le(−) [36, 45–47]. For females who belong to the Se(+)Le( +) genotype and therefore have functional FUT2 and FUT3 enzymes, all types of FucOS can be found in their milk. The Se(−)Le( +) women produce milk containing FucOS with *α*1,3 and *α*1,4 linkages such as lacto-N-fucopentaose (LNFP) II, LNFP III, and 3-fucosyllactose (FL). Mothers with the Se(+)Le(−) genotype can synthesize LNFP I, LNFP III, 2′-FL, and 3-FL. Those identified as Se(−)Le(−) are capable of producing FucOS with *α*1,3 bonds such as LNFP III, LNFP V, and 3-FL. However, under some circumstances the biosynthesis of FusOS cannot be perfectly elucidated by the expression of Se and Le genes, implying that there might be an unknown FUT taking part in this process or an unidentified synthetic pathway independent from FUT [19, 44, 48–51].

As for biosynthesis of SiaOS, two genes namely Le and ABH are implicated in this process. Low levels of SiaOS are observed in the milk of mothers with the ABH(−)Le(−) genotype, while those representing the ABH(+)Le(+) genotype can express high levels of SiaOS such as disialyllacto-*N*-tetraose (DSLNT), LS-tetrasaccharide (LSTa), 3′-sialyllactose (SL), and 6′-SL [52–54].

Moreover, biosynthesis of core OS contributes to increased variability of HMO structures in human milk. There are four glycosyltransferases activated in this course: *β*3-galactosyltransferases and *β*4-galactosyltransferases are responsible for Gal relocation, while *β*1,3-*N*-acetylglucosaminyltransferase (iGnT) and *β*1,6-*N*-acetylglucosaminyltransferase (IGnT) are involved in the GlcNAc transfer [55, 56].

Of note, besides various genes participating in HMOs biosynthesis, it is believed that there are other factors also influence endogenous synthesis of HMO. Though total HMO concentration decreased substantially over the course of lactation [35], a more significant decrease in HMO would occur in the effect of the seasonal changes and certain nutritional conditions of the mother. In Gambia, lactating mothers who nursed their children during the wet season produce milk with lower HMO concentration in comparison to those nursing in the dry season when the food is more plentiful and energy intake is higher [57]. Besides, a Canadian study suggests that other seasonal factors such as climate, sunlight, and allergen exposures might influence HMO synthesis in Canadian population [45]. Similarly, the HMO composition in breast milk may be changed when mothers are supplemented with a mixture of probiotics during late stages of pregnancy. Seppo et al. showed that the concentrations of 3-FL and 3′-SL were significantly higher in the colostrum of mothers who received probiotic supplementation than in control participants; however, the total concentration of HMOs still decreased in colostrum from the mothers in the probiotic supplementation group due to the lower levels of difucosyllacto-*N*-hexaose, lacto-*N*-tetraose (LNT), LNFP I, and 6′-SL [58]. Another study also indicated the positive association between SiaOS concentration and vitamin A intake [59], while a lipid-based nutrient supplement showed no effect on HMO concentration [60]. When it comes to the effect of maternal age, weight, body mass index, and parity on the endogenous synthesis of HMO, there is a contradiction between different studies [45, 47, 57, 61]. In brief, studies mentioned above all suggest that wide variability of HMOs due to the genes polymorphism and environmental condition may have different effects on gut microbiota development, infant health, and disease risk.

### Metabolism of HMOs

HMOs are resistant against an infant’s digestive enzymes and can remain their special structural configuration through the proximal intestine, which has been affirmed by several clinical studies [54, 62–64]. Then they would reach the distal intestine serving as a substrate fermented by specific intestinal microbiota, such as *Firmicutes*, *Proteobacteria*, and especially *Bifidobacterium* spp. [39, 63, 65–67]. In particular, HMOs degradation mediated by *Bifidobacterium* spp. can be divided into two strategies. The first approach is initialed by the importation of complete HMOs into the cytoplasm through adenosine triphosphate binding cassette (ABC) transporter, which will be hydrolyzed by intracellular glycosidases, while the other one depends on cell wall-anchored secretory glycosyl hydrolases (GHs), which hydrolyze HMOs and release monosaccharides and disaccharides [68, 69]. For example, *B. bifidum* uses extracellular hydrolases releasing LNB which is the core structure of type-1 HMO, while *B. infantis* and *B. breve* use oligosaccharide transporters [70]. However, the HMOs degradation pattern of *B. longum* depends on strains and the existence of lactam-N-biogenase (LnbX). LnbX-negative *B. longum* utilizes oligosaccharide transporters to assimilate HMO derivatives internally, whereas LnbX-positive *B. longum* utilizes extracellular hydrolases [71]. Furthermore, depending on the consumption of certain HMOs, some microbes that are capable of catabolizing HMOs will obtain predomination over others [72, 73], which function as a probiotic shaping the infant’s intestinal microbiota [74, 75]. For instance, the predominant presence of intestinal bacteria from *Bifidobacterium* spp. has been related to human milk with a higher content of sialyllacto-*N*-tetraose b (LSTb), monofucosyllacto-N-hexaose (MFLNH)- III, DSLNT, LNFP I, LNFP III, and LNFP V; whereas 2’-FL, lacto-N-hexaose (LNH) and two of its isomers found in human milk benefited the growth of *Bacteroides* spp. [63, 66, 76].

After ingested, about 99% of HMO reach the intestine and nearly 45% of them are fermented by intestinal microbiota, while 1–4% and 40–50% of total HMOs ingested are excreted in the urine and feces, respectively [54, 62]. The remaining 1% are absorbed at concentrations of 0.10–0.20 g/L, resulting in plasma concentration of 0.01–0.10 mg/L [20, 77, 78]. According to Goehring et al. [54] and Vazquez et al. [78], 2′-FL and lacto-*N*-neotetraose (LNnT), which are smaller molecular weight HMOs, are absorbed quickly into bloodstream and excreted in the urine without metabolism, suggesting that certain HMOs in urine may reflect the mother’s secretor/nonsecretor status, whereas HMOs with fecal excretion varies remarkably correlating with infant’s different intestinal microbiota [62]. Notably, some novel HMOs that not related to common HMOs are found in infant's urine and feces due to microbial metabolism [62], showing differences in the metabolism patterns of HMOs. Furthermore, in contrast to traditional cognition that the infant has contact with mother’s HMOs through postnatal feeding, recent studies revealed that the fetus might have been already exposed to HMOs in utero given the presence of HMOs in mothers body fluid during pregnancy and in amniotic fluid at delivery [79, 80].

## Microbiota

HMOs once ingested begin to interact with various microbes including bacteria, viruses, protozoan parasites, and fungi inside the infant body leading to a series of constructive effects indirectly [81]. Therefore, a better understanding of neonate microbiota will definitely advance our insight into the positive role of HMOs.

After the process of giving birth, bacteria colonize in the relatively sterile gastrointestinal tract of the newborn rapidly, which marks the beginning of the highly complex formation of the microbiota [82, 83]. Therefore, the first year of newborn’s life is crucial for the establishment of the intestinal microbiome, which underlies the folate production, reduction of allergic diseases, increased immune responses to vaccinations, synthesis of essential vitamins and other molecules that serve as modulators of physiological responses and are used as energy source by the intestinal epithelium [84–89]. The earliest gut microbiome is characterized by the colonization of facultative anaerobes, such as *streptococci*, *enterococci*, and *staphylococci* [82]. As the main component of infant diet, the wide presence of HMO in human milk act as one of the most vital factors shaping the latter gut microbiome that represented by *Bacteroides* spp., *Clostridium* spp., and especially *Bifidobacterium* spp. with up to 90% of total microbes within the first 3 months of the baby’s life [90, 91].

*Bifidobacteria* are gram-positive and heterofermentative obligate anaerobes which are among the first bacteria to inhabit human digestive tracts [92], with 78 species and 10 subspecies classified to date [93–96]. About ten (sub)species were isolated from human feces and certain species seem to be frequently found in the infant gut, such as *B. breve* and *B. infantis* [97–100], while other species, such as *B. longum*, *B. pseudocatenulatum*, and *B. bifidum* are likely to inhabit both in the infant and adult gut microbiome [101, 102]. Besides, *Bifidobacterium* spp. remain the dominant bacteria in infant gut microbiota during breast-feeding, yet the relative abundances quickly decline after weaning [98, 103], during when the compositional change takes place at species level [104], indicating the direct correlation between HMOs and developing infant microbiota. According to several studies, a significant decline in fecal HMOs was positively correlated with high levels of HMO-consuming *bifidobacteria* [76, 97]; furthermore, a specific *β*-galactosidase from *B. longum* was applied to catabolize HMOs in vitro, whose catabolized fragments were found to match with compounds identified in infant fecal samples [105]. Subsequent studies focus on the consumption of HMOs by *Bifidobacterium* spp. As Ward et al. reported, *B. infantis* ATCC 15697 was observed to use HMOs as a sole carbon source [106], and studies conducted by Garrido et al. showed a similar result that isolates of *B. infantis* grew well on pooled HMOs and individual HMO sugars while some examined strains of *B. bifidum* could not grow with 2′-FL and 6′-SL as a sole carbon source [68]. Notably, while HMOs are mainly consumed by *bifidobacteria*, it is undoubted that certain isolates of *bifidobacteria* have stronger capability to ferment HMOs, meanwhile, some types of HMO are more frequently assimilated by *bifidobacteria* than others. Therefore, accumulating studies have tried to figure out the mechanisms of how *bifidobacteria* catabolize HMOs and revealed that the consumption of HMO is well conserved among *B. infantis* strains, fermenting all classes of HMO [68, 107, 108]. Take *B. infantis* ATCC 15697 for example, this strain of *B. infantis* can utilize several types of HMO including sialylated and fucosylated molecules [108]. The genome sequence of *B. infantis* ATCC 15697 showed a great number of HMO-utilization genes located in a specific segment of the genome, namely HMO cluster I [109], whose expression led to up-regulation of GHs [110] and family 1 solute binding proteins (SBPs) that is a part of ABC transporters for HMOs [111]. The process of HMO consumption begins with the SBPs-mediated importation of intact HMOs inside the cytoplasm, then HMOs were assimilated intracellularly by several GHs releasing large quantities of lactic and acetic acid, who play an important role in modulating intestinal physiology and protecting infant gut from pathogen colonization [112–114]. Besides, RNA-seq transcriptomic analysis also revealed that 6’-SL, lacto-N-tetraose, and LNnT could induce the expression of HMO-utilization genes in the HMO cluster I, whereas alternate gene clusters other than HMO cluster I seem to be induced to utilize Fuc during growth in presence of 2’-FL and 3-FL [68]. In brief, all these analyses are crucial for further studies to deepen the understanding of regulatory networks behind HMO consumption and of how HMO consumption is associated with the gut microbiota establishment in breast-fed infants, underlying the design of novel HMO analogs targeting selected beneficial *bifidobacteria* [70, 115, 116].

Apart from promoting the growth of beneficial bacteria, HMO-mediated anti-bacterial effects have been observed in *Campylobacter jejuni* [117], *Escherichia coli* pathogenesis [31, 118], and *Listeria monocytogenes* [119–121]. HMOs can also act as antiviral agents to provide protection against a number of viral pathogens, such as norovirus [122, 123], rotavirus [124], and respiratory virus [125, 126], through several mechanisms. Besides, despite the limited studies which assess the activity of HMOs against protozoan parasites and fungal species, the findings from these studies demonstrated that HMOs can reduce infection by *Entamoeba histolytica* [127] and *Candida albicans* [128]*.* In conclusion, HMOs have a great influence on infant microbiota, indicating their potential as novel candidates for further developments in food additive to infant formula milk and therapeutics targeting pathogenic infection.

## Effects of HMO on infant gut microbiota

Diverse functions of HMOs have been demonstrated, such as regulating microbiota composition, protecting against pathogen adhesion and infection, and modulating epithelial cell response. In the section below, we will focus on the functions that attribute to HMOs and the mechanism underlying the beneficial effects of HMOs.

### Effects of HMO on microbiota composition

**Fig. 3:**
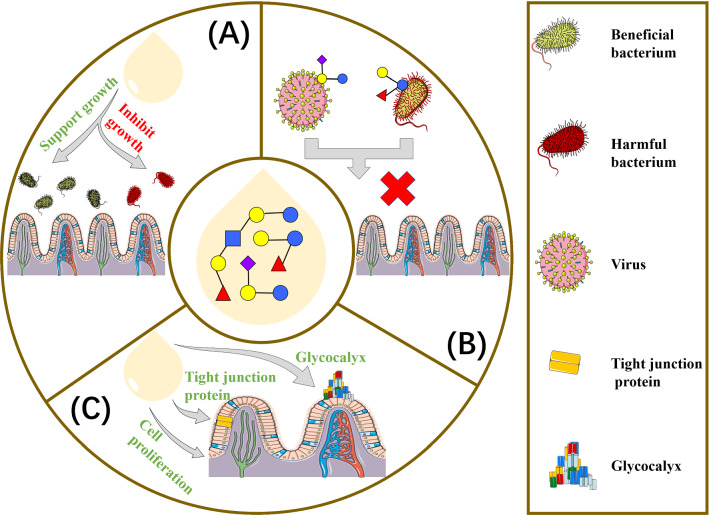
Schematic summary of main effects of HMOs. **A** HMOs stimulate growth of beneficial bactria, such as *Bififidobacteria*, and inhibit growth of harmful bacteria to regulate gut microbiota composition [132]. **B** HMOs serve as pathogen binding decoy receptors to prevent pathogens from binding to epithelial cell receptors [149]. **C** HMOs alter glycocalyx [171], influence epithelial cell proliferation [168] and modulate tight junction protein expression [167], thereby reducing permeability of the gut barrier

HMOs have an important influence on bacteria colonization in the intestine that is necessary for infant health. In early life, 10^14^ bacteria colonized the intestine [129]. The first year of infant life is critical for intestinal microbiome establishment, and infant diet is of importance for gut microbiome development [90]. HMOs are not digested in the top half of the gastrointestinal tract of infants, due to the lack of GHs and intestinal membrane transporters [130, 131]. As a consequence of high concentration, HMOs can reach both the small and large intestine, where they serve as substrates for resident microbes, affecting the composition and activity of the gastrointestinal microbiota [132] (Fig. 3A). HMOs are specifically known to support the growth of beneficial microorganisms, such as *Bifidobacterium* [133], which is generally calculating for 50–90% of the total bacterial population detected in the feces of breastfed infants [134]. Genomic analysis of particular infant-derived *Bifidobacterial* strains has revealed that aggregation of transporters, GHs, and carbohydrate-binding proteins contributes to the degradation of HMOs [135]. The expression of HMOs-degrading enzymes is mainly limited to *B. breve*, *B. bifidum*, *B. longum*, and *B. infantis* [136, 137]. Besides, *Bifidobacteria* and *Lactobacilli* express sialidases and fucosidases to cleave Sia and Fuc, respectively, indicating the coevolution of these species and HMOs [107].

*Bifidobacterium* also have an impact on other microorganisms composition. Asakuma et al. found that *B. bifidum* can secrete GHs to degrade HMOs extracellularly, and then leave metabolized sugars outside the cells, which are utilized by other bacteria to produce short chain fatty acids (SCFAs) such as butyrate and propionate [138]. Butyric acid and propionic acid are essential for intestinal health given that they can interact with host epithelial cells to stimulate mucin release, increase mucosal blood flow, and regulate immunity [139, 140]. *E. hallii*, which is a common member of the adult gut microbiota, cannot grow by using Fuc. However, when cocultured with *B. infantis*, it can utilize 1,2-propanediol (1,2-PD), which is produced by *B. infantis* through resolving Fuc, revealing a trophic interaction between *E. hallii* and *B. infantis* [141].

Apart from the capability of HMOs to modulate the composition of *Bifidobacteria* in the intestinal tract, they can directly interact with other bacteria and affect the distribution of gut microbes. 2'-FL, 3-FL, 3'-SL, 6'-SL, and lactodifucotetraoseare (LDFT) are metabolized by many *Bacteroides* [142]. Besides, HMO-derivatives LNB and Lacto-*N*-Triose II can be used by *L. casei* [143]. In conclusion, though the species of gut microbes except bifidobacteria have low capacity, they still play an important role in intestinal homeostasis of breastfed infants [144].

Additionally, HMOs can pass gut-barrier so that they have an effect outside the intestines [145]. HMOs have been demonstrated to activate G protein-coupled receptors (GPCRs) which can influence almost every physiological function, such as development, taste, olfaction, regulating heart rate, hormone signaling, and neurotransmission [146] in two ways: one pathway is direct activation by 6′-SL and LNT, the second is increased production of kynurenic acid (KYNA) by the microbiota which in turn activates GPR35 [147].

### Effects of HMO on preventing pathogen infection

Several bacteria, viruses, fungi, and protozoan parasite need to adhere to the glycocalyx (the carbohydrate-rich layer coating epithelial cells) first to invade the host and cause diseases [148], while HMOs can prevent the infection by acting as soluble decoy receptors, which combine with pathogens to avoid them from binding to epithelial cell surface receptors, therefore, the pathogens would pass gastrointestinal tract harmlessly [149] (Fig. 3B).

#### Bacterial infection

*Campylobacter jejuni* seems to be one of the most common causes of diarrhea which leads to infants death [150]. 2'-FL acts as a soluble decoy receptor for *C. jejuni*, reducing the colonization of *C. jejuni* by 80% [117, 151]. Enteropathogenic *Escherichia coli* (EPEC) can cause serious diarrheal disease leading to high mortality rates in infants. A significant reduction of the pathogenic colonization is observed in cultured epithelial cells by pre-incubating EPEC with mixed HMO components [118]. 2′-FL and LNFP I not only reduce the adhesion of pathogens but also decrease pathogenicity by binding to heat-labile enterotoxin type 1 [31]. It also plays a role in immunity and urinary system. Lately, He et al. found that lipopolysaccharide-mediated inflammation was directly inhibited by 2'-FL during the process of enterotoxigenic *Escherichia coli* (ETEC) invading T84 and H4 intestinal epithelial cells [152]. Similarly, uropathogenic *Escherichia coli* (UPEC) would be prevented from attaching to epithelial cell monolayers in the presence of 15 mg/mL HMOs, which delay the p38 MAPK and p65 NF-κB signaling pathways [153].

Aside from decreasing the adhesion and invasion of pathogens, HMOs can modify the gene expression of epithelial surface and inhibit the growth of pathogens to reduce their infection. After pre-incubating with HMOs, genes of Caco-2Bbe gut cells that mediate the adhesion between intestinal epithelial cells and *L. monocytogenes* would be downregulated due to the activation of unfolded protein response and eIF2 signaling [119]. Another study revealed that growth and biofilm formation of Group B *Streptococci* (GBS) can be modulated by HMOs as well. In specific, the concentration of HMOs between 1–2 mg/L delays the growth of GBS up to 96–98%, and LNT and lacto-N-difucohexaose (LNDFH)-I showed the highest capability of inhibition [121]. Besides, the combination of HMOs and vancomycin or ciprofloxacin will improve the curative effect of these antibiotics [120, 154].

#### Viral infections

HMOs can improve infant resistance to two fatal gastrointestinal infections caused by rotavirus and norovirus [124, 155]. Mechanical studies revealed that HMOs provided protection against viral infections by mimicking receptor sites to prevent viruses from entering host cells [156] and stimulate immunity through γ-interferon and IL-10 expression to decrease virulence [157].

Most recently, 2′-FL, 3′-SL, and 6′-SL were demonstrated to have a notable antiviral activity against G1P[8] and G2P[4] rotavirus. 2′-FL significantly inhibited G1P[8] rotavirus infection, while a conjugate of 3′-SL and 6′-SL had the strongest ability to inhibit G2P[4] rotavirus infection [124]. However, HMOs cannot inhibit all kinds of rotavirus infections, such as neonatal rotavirus G10P[11], it has a dose-dependent enhancement in infectivity with the increased concentration of LNT and LNnT [155].

HMOs have also been shown to function as antiviral agents to prevent norovirus infection. Histo-blood group antigens (HBGAs), which function as key binding sites for norovirus adhesion, are carbohydrate epitopes not only present in the surface of red blood cells, but also in mucosal epithelium of the gastrointestinal tracts, genitourinary tracts, and the respiratory tubes [28]. They also act as free oligosaccharide fluids in the physiological system [158, 159]. Norovirus can bind to high-mass HMOs containing abundant α-fucose due to the similar structure to HBGAs [122], resulting in the reduced infection of breastfed infants. Similarly, Fuc and Glc were the fermentation products of 2’-FL and 3’-FL, which can connect to GI.1, GII.17, and GII.10 noroviruses by interacting with amino acids expressed in noroviruses to prevent norovirus from binding to HBGAs [123, 160].

In addition to preventing the gut virus, HMOs also can inhibit respiratory virus infections [161]. 2′-FL has been shown to reduce viral load of respiratory syncytial virus [162]. And further research showed that the 2′-FL is possible to enhance innate and adaptive immunity in influenza-specific murine model [125].

#### Protozoan parasite infections

Beyond acting as an inhibitor of bacterial and viral pathogens, HMOs significantly prevent protozoan parasite infections as well. *Entamoeba histolytica* is an anaerobic amoebozoan which causes 55,000 deaths worldwide every year [163]. An in vitro study demonstrated that LNT which contains the terminal Gal structure can act as soluble decoy receptors to prevent *Entamoeba histolytica* from attaching to intestinal epithelial HT-29 cells [127].

#### Fungal infection

The impact of intestinal fungus on infant health is especially significant in early gestational age. For example, the invasion rate of systemic candidiasis in infants approximately reaches 10% and the mortality rate is about 20% [164]. A recent study showed that HMOs downregulated ALS3 that encoded the *C. albicans* hyphal-specific adhesion, and nascent hyphae expression, resulting in the reduced adhesion between *C. albicans* and epithelial cells at early infection phase. Additionally, the intestinal epithelial cell binding sites on the surface of *C. albicans* are blocked by HMOs as well [128].

### Effects of HMOs on modulating epithelial cell responses

HMOs not only influence microbes intensely but also have a direct effect on intestinal epithelial cells (Fig. 3C). The intestinal epithelium covering the small intestine and colon is regarded as a paramount part of innate immunity, serving as a physical and speed limit barrier between intestinal cavity and circulatory system [165]. The tight junctions connecting epithelial cells determine the permeability of the epithelium, which is known as permselective barrier. It modulates the process of macromolecules and ions passing the pore and leak ways, avoiding the absorption of harmful microbes and compounds, and regulating the transportation of electrolytes and nutrients [166]. HMOs can modulate the expression of tight junction protein, thereby decrease the permeability and enhance the barrier effect of the epithelium. 2-′FL, 6-′SL, or LNnT can arrest G2/M cell-cycle of HT-29 and Caco-2Bbe, which belongs to small intestinal cell lines, to inhibit the proliferation of HT-29 and Caco-2Bbe in preconfluent phase, leading to the maturation of HT-29 and Caco-2Bbe given that the differentiation and proliferation are inversely proportional in preconfluent cultures; besides, the high concentration of LNnT and 2′-FL can also enhance barrier function and promote digestion [167]. Further study showed that whether HT-29 and Caco-2Bbe were treated with individual or compositional 2′-FL, 3′-SL and 6′-SL, the proliferation of them would be reduced and the differentiation would be increased in preconfluent transformation, all of which can promote the maturation of HT-29 and Caco-2Bbe cell lines [168]. Furthermore, HMOs can upregulate the expression of Muc2 which is the predominant form of mucin in the small intestine leading to descended bacterial adhesion and permeability of the intestinal epithelium [169].

The unproper development of glycocalyx on neonatal gut epithelium will disorder the gastrointestinal system [170]. A recent study indicated that 2′‐FL and 3′‐FL promoted glycocalyx development in a structure‐dependent fashion and gut barrier will be enhanced subsequently [171]. Besides, the transformation from sialylation to fucosylation benefits the maturation of intestinal epithelium [172], which means HMOs can regulate intestinal epithelial cells through modulation of intestinal glycome [173]. Angeloni et al. discovered that the expression of sialyltransferases ST3Gal1, ST3Gal2, and ST3Gal4 would be decreased in the presence of 3′‐FL, leading to the reduction of *α*2-3-, *α*2-6-sialylation on Caco-2Bbe surface, which results in the reduced adhesion of *E. coli* by 50% [174].

HMOs also show indirect effects on epithelium after fermented by *B. infantis*. A study has reported that the conditioned media of *B. infantis* (BCM) enhanced expression of occludin and junctional adhesion molecule in either HT-29 or Caco-2Bbe, which can improve intestinal barrier function [175]. BCM also increased claudin-1 protein expression, by which the gut barrier was strengthened [176]. And the BCM might prevent IL-1b stimulation to protect Caco-2Bbe through NF-κB pathway as well [177].

## Conclusion and future perspectives

HMOs are complex carbohydrates synthesized in breast gland which are abundant in human milk. Different kinds of HMOs directly or indirectly modulate the infant’s physiological systems by regulating microbial composition, preventing pathogens adhesion and invasion, and regulating intestinal epithelial cell response.

Currently, HMOs have been synthesized artificially as additives in infant milk formulations for the infants who cannot be fed with breast milk to support their growth and provide protection against different diseases which have a high morbidity in babys’ early years [171, 178]. Some studies have shown the superior assimilation and toleration of 2′-FL and LNnT by infants [179, 180], meanwhile other HMOs still have challenges in expensive synthesis. Given that, the European Union and the United State consider 2′-FL and LNnT are qualified to be used in infant formula [181]. A clinical study showed that formula with 2’-FL can inhibit inflammatory cytokine production and the results of the formula group are similar to the breastfed group [182]. The other study revealed that the formula 2′-FL and LNnT would keep infants healthy whose parents have respiratory tract infections and bronchitis [180]. Recently, it was found that the addition of 2′-FL and LNnT to infant formula would shift the microbiota toward the microbiota of breastfed babies, which would increase the quantity of *Bifidobacteria* and decrease the number of *Clostridium difficile* [183].

The application of HMOs in therapeutic area has been reported in recent years. For instance, HMOs have therapeutic potential in food allergies. HMO supplementation study was conducted in an ovalbumin sensitized mouse model consuming 2′-FL and 6′-FL. As a consequence, 2′-FL and 6′-FL would indirectly stabilize mast cells by inducing expression of T regulatory cells, and activate the IL-10(+) regulatory cells to reduce the symptoms of food allergy [184]. Especially, 6′-FL can suppress the immune system greatly by decreasing inflammatory factors and chemokines, which inhibit inflammatory cells from flocking in the intestine [184]. Analogously, FUT2-dependent breast milk oligosaccharides reduced the occurrence of IgE-associated disease and IgE-associated eczema in cesarean section born infants [185]. In contrast to these findings, pro-inflammatory effect of 3′-SL was reported in a mesenteric lymph node CD11(+) dendritic cells exposed to 3′-SL, which can generate cytokines that increase the quantity of Th1 and Th17 immune cell [186]. Besides, clinical studies have confirmed that HMOs contribute to the positive effects of human milk against necrotizing enterocolitis (NEC) which is a fatal gastrointestinal disease in very low birth weight (VLBW) infants [187]. DSLNT in breast milk could be used to prevent NEC in formula-fed infants, and its concentration in the mother’s milk could act as a potential non-invasive marker to identify whether infants are at risk of NEC [187], while another study including 96 mothers and 106 VLBW infants demonstrated a contradictory result that DSLNT was not significantly associated with NEC [178]. 2′-FL, 3′-SL, 6′-SL, and LNnT also have a protective effect on the development of autoimmune diseases such as type-1 diabetes (T1D) which is caused by autoimmune destruction of insulin-producing *β* cells of the pancreas. Animal research revealed that HMOs were prone to balance Th1/Th17 immune responses of non-obese diabetic (NOD)-mice, which would reduce T1D occurrence rate and inhibit pancreatic insulitis progress [125]. Metabolic products of HMOs have been demonstrated in field of cognition development [67]. An animal study showed the dietary 2′-FL improved cognitive abilities, learning and memory in rodents [188]. Furthermore, 3’-SL and 6’-SL were able to supported normal microbial communities and behavioral responses in stressor-exposed mice to prevent stressor-induced effect, and the result revealed the evidence of gut microbiota-brain axis [189].

HMOs not merely affect infants, but also have an influence on adults. 2′‐FL and LNnT can change the microorganisms composition of adults, which increasing the amount of *Bifidobacterium* and *Actinobacteria*, and decreasing *Proteobacteria* and *Firmicute* [190]. Another study revealed that Caco-2Bbe treated with 2′-FL for 3 weeks showed reduced permeability of monolayers, and tight junction proteins, such as claudin-5 and claudin-8 were upregulated, which strengthen the gut barrier in adults [191].

In conclusion, HMO plays a special role in the prevention and treatment of diseases, thereby maintaining the health of infants and adults. Therefore, the prospect of HMO will be exciting, both for the prevention of single disease and multiple combined diseases.

## Data Availability

Not applicable.

## Abbreviations

WHO: World Health Organization
HMOs: Human milk oligosaccharides
OS: Oligosaccharides
GlcNAc: *N*-acetylglucosamine
Sia: Sialic acid
Fuc: l-fucose
Gal: d-galactose
Glc: d-glucose
LNB: Lacto-*N*-biose
FucOS: Fucosylated OS
SiaOS: Sialylated OS
Se: Secretor
Le: Lewis
FUT2: α1-2-Fucosyltransferase enzyme
FUT3: α1–3/4-Fucosyltransferase
LNFP: Lacto-*N*-fucopentaose
FL: Fucosyllactose
DSLNT: Disialyllacto-*N*-tetraose
LSTa: LS-tetrasaccharide
SL: Sialyllactose
iGnT: β1,3-*N*-acetylglucosaminyltransferase
IGnT: β1,6-*N*-acetylglucosaminyltransferase
LNT: Lacto-*N*-tetraose
ABC: Adenosine triphosphate binding cassette
GHs: Glycosyl hydrolases
LnbX: Lactam-*N*-biogenase
LSTb: Sialyllacto-*N*-tetraose b
MFLNH: Monofucosyllacto-*N*-hexaose
LNH: Acto-*N*-hexaose
LNnT: Lacto-*N*-neotetraose
SBPs: Family 1 solute binding proteins
SCFAs: Short chain fatty acids
1,2-PD: 1,2-Propanediol
LDFT: Lactodifucotetraoseare
GPCRs: G protein-coupled receptors
KYNA: Kynurenic acid
EPEC: Enteropathogenic *Escherichia coli*
ETEC: Enterotoxigenic *Escherichia coli*
UPEC: Uropathogenic *Escherichia coli*
GBS: Group B *Streptococci*
LNDFH: Lacto-*N*-difucohexaose
HBGAs: Histo-blood group antigens
BCM: Conditioned media of *B. infantis*
NEC: Necrotizing enterocolitis
VLBW: Very low birth weight
LNDH I: Lacto-*N*-difucohexaose
T1D: Type-1 diabetes
NOD: Non-obese diabetic
