# Correction to: Gold standard for nutrition: a review of human milk oligosaccharide and its effects on infant gut microbiota

**DOI:** 10.1186/s12934-021-01612-4

**Published:** 2021-07-21

**Authors:** Shunhao Zhang, Tianle Li, Jing Xie, Demao Zhang, Caixia Pi, Lingyun Zhou, Wenbin Yang

**Affiliations:** 1 State Key Laboratory of Oral Disease, National Clinical Research Center for Oral Disease, West China Hospital of Stomatology, Sichuan University, Chengdu, 610041 Sichuan China; 2 Center of Infectious Diseases, West China Hospital of Sichuan University, No. 37 Guoxue Alley, Wuhou District, Chengdu, 610041 China; 3 State Key Laboratory of Oral Diseases, National Clinical Research Center for Oral Diseases, Department of Oral and Maxillofacial Surgery, Department of Medical Affairs, West China Hospital of Stomatology, Sichuan University, No. 14, Section 3, South Renmin Road, Chengdu, 610041 Sichuan China

## Correction to: Microb Cell Fact (2021) 20:108 https://doi.org/10.1186/s12934-021-01599-y

Following publication of the original article [1], the authors identified a problem with the authorship. In the original authorship, Tianle Li had been erroneously detailed as the second author. However, as Shunhao Zhang and Tianle Li contributed equally to this work, Tianle Li should in fact be regarded as a co-first author.

The authorship has been updated in the original article and the correct authorship may be seen in this correction.

The authors apologize for this mistake and any inconvenience caused. All authors agree to this correction of authorship.
